# Serological Surveillance Development for Tropical Infectious Diseases Using Simultaneous Microsphere-Based Multiplex Assays and Finite Mixture Models

**DOI:** 10.1371/journal.pntd.0003040

**Published:** 2014-07-31

**Authors:** Yoshito Fujii, Satoshi Kaneko, Samson Muuo Nzou, Matilu Mwau, Sammy M. Njenga, Chihiro Tanigawa, James Kimotho, Anne Wanjiru Mwangi, Ibrahim Kiche, Sohkichi Matsumoto, Mamiko Niki, Mayuko Osada-Oka, Yoshio Ichinose, Manabu Inoue, Makoto Itoh, Hiroshi Tachibana, Kazunari Ishii, Takafumi Tsuboi, Lay Myint Yoshida, Dinesh Mondal, Rashidul Haque, Shinjiro Hamano, Mwatasa Changoma, Tomonori Hoshi, Ken-ichi Kamo, Mohamed Karama, Masashi Miura, Kenji Hirayama

**Affiliations:** 1 Department of Eco-Epidemiology, Institute of Tropical Medicine, Nagasaki University (NUITM), Nagasaki, Japan; 2 Graduate School of International Health Development, Nagasaki University, Nagasaki, Japan; 3 Nagasaki University Institute of Tropical Medicine (NUITM)- Kenya Medical Research Institute (KEMRI), Nairobi, Kenya; 4 Centre for Infectious and Parasitic Diseases Control Research, Kenya Medical Research Institute (KEMRI), Busia, Kenya; 5 Eastern & Southern Africa Centre of International Parasite Control (ESACIPAC), Kenya Medical Research Institute (KEMRI), Nairobi, Kenya; 6 Production Department, Kenya Medical Research Institute (KEMRI), Nairobi, Kenya; 7 Thomas Odhiambo Campus, Mbita, International Center of Insect Physiology and Ecology (ICIPE), Mbita, Kenya; 8 Department of Bacteriology, Osaka City University Graduate School of Medicine, Osaka, Japan; 9 Food Hygiene and Environmental Health Division of Applied Life Science, Graduate School of Life and Environmental Sciences, Kyoto Prefectural University, Kyoto, Japan; 10 Kenya Research Station, Nagasaki University, Nagasaki, Japan; 11 Department of Infection and Immunology, Aichi Medical University School of Medicine, Nagakute, Aichi, Japan; 12 Department of Infectious Diseases, Tokai University School of Medicine, Kanagawa, Japan; 13 Department of Microbiology and Immunology, Faculty of Medicine, Fukuoka University, Fukuoka, Japan; 14 Division of Malaria Research, Proteo-Science Center, Ehime University, Ehime, Japan; 15 Department of Paediatric Infectious Diseases, Institute of Tropical Medicine, Nagasaki University, Nagasaki, Japan; 16 International Center for Diarrheal Disease Research (ICDDR, B), Dhaka, Bangladesh; 17 Department of Parasitology, Institute of Tropical Medicine, Nagasaki University, Nagasaki, Japan; 18 Department of Liberal Arts and Sciences, Sapporo Medical University, Sapporo, Japan; 19 Centre of Public Health Research, Kenya Medical Research Institute (KEMRI), Nairobi, Kenya; 20 Department of Immunogenetics, Institute of Tropical Medicine, Nagasaki University, Nagasaki, Japan; Centers for Disease Control and Prevention, United States of America

## Abstract

**Background:**

A strategy to combat infectious diseases, including neglected tropical diseases (NTDs), will depend on the development of reliable epidemiological surveillance methods. To establish a simple and practical seroprevalence detection system, we developed a microsphere-based multiplex immunoassay system and evaluated utility using samples obtained in Kenya.

**Methods:**

We developed a microsphere-based immuno-assay system to simultaneously measure the individual levels of plasma antibody (IgG) against 8 antigens derived from 6 pathogens: *Entamoeba histolytica* (C-IgL), *Leishmania donovani* (KRP42), *Toxoplasma gondii* (SAG1), *Wuchereria bancrofti* (SXP1), HIV (gag, gp120 and gp41), and *Vibrio cholerae* (cholera toxin). The assay system was validated using appropriate control samples. The assay system was applied for 3411 blood samples collected from the general population randomly selected from two health and demographic surveillance system (HDSS) cohorts in the coastal and western regions of Kenya. The immunoassay values distribution for each antigen was mathematically defined by a finite mixture model, and cut-off values were optimized.

**Findings:**

Sensitivities and specificities for each antigen ranged between 71 and 100%. Seroprevalences for each pathogen from the Kwale and Mbita HDSS sites (respectively) were as follows: HIV, 3.0% and 20.1%; *L. donovani*, 12.6% and 17.3%; *E. histolytica*, 12.8% and 16.6%; and *T. gondii*, 30.9% and 28.2%. Seroprevalences of *W. bancrofti* and *V. cholerae* showed relatively high figures, especially among children. The results might be affected by immunological cross reactions between *W. bancrofti*-SXP1 and other parasitic infections; and cholera toxin and the enterotoxigenic *E. coli* (ETEC), respectively.

**Interpretation:**

A microsphere-based multi-serological assay system can provide an opportunity to comprehensively grasp epidemiological features for NTDs. By adding pathogens and antigens of interest, optimized made-to-order high-quality programs can be established to utilize limited resources to effectively control NTDs in Africa.

## Author Summary

Monitoring the distribution of neglected tropical diseases (NTDs) is a key to controlling their spread in Africa. Currently, such surveillance is conducted independently for each NTD. To tackle this problem, we developed a microsphere-based system to permit simultaneous measurement of IgG antibody levels for antigens from six infectious diseases: *Entamoeba histolytica*, *Leishmania donovani*, *Toxoplasma gondii*, *Wuchereria bancrofti*, HIV, and *Vibrio cholerae*. Using this system, we conducted a serological survey using two health and demographic surveillance system (HDSS) areas in coastal and western Kenya. We randomly selected 4,600 individuals according to sex and age group, of whom 3411 agreed to participate in the study. Mathematical analyses of the distributions of the participants' reactivity to each antigen and the reactivity of the sero-positive and -negative controls indicated that this system could be used to monitor infections, especially, those associated with HIV, filariasis, toxoplasmosis, leishmaniasis, and amebiasis. For the practical development and eventual implementation of actual programs in Africa, pathogens and antigens of interest can be added to optimize made-to-order monitoring programs.

## Introduction

Combating infectious diseases, including neglected tropical diseases (NTDs), among the poorest segment of the population is a major concern of the international community. In sub-Saharan Africa, the impact of NTDs as a group is comparable to that of malaria and tuberculosis; however, NTDs collectively receive less attention and research funding than human immunodeficiency virus/acquired immunodeficiency syndrome (HIV/AIDS), tuberculosis, or malaria, despite the fact that NTDs affect an estimated one billion people in tropical and subtropical climates [1]. The poor can be afflicted by more than one NTD. According to the World Health Organization, approximately 74% of the affected population simultaneously harbors two NTDs; 18% three; 13% four; 9% five; 11% six; and 8% seven or more NTDs [2]. Furthermore, most NTDs have a geographical distribution similar to that of HIV, malaria, and tuberculosis [3]. The population at risk for NTDs also is at risk for these life-threatening infectious diseases.

Although prevalence surveys of NTDs used for monitoring temporal and geographical distributions of each disease are important from a public health point of view, such surveys are limited by technical issues. For instance, diagnosis must be performed independently for each disease. If infectious diseases could be concurrently detected, the cost and effectiveness of diagnosis may improve; and timely data on the prevalence of NTDs and other infectious diseases may become more readily available [4].

Microsphere-based multi-serological assays permit the simultaneous measurement of many different analytes in a small sample volume [5]. Technically, such assay systems apply the same concepts as that of flow cytometry, but unlike flow cytometry, the microsphere-based assays use 500 unique dye mixtures to identify individual microspheres. Theoretically, a multiplexed technique can concurrently run up to 500 different assays. In practice, such methods already are being used for screening different serotypes of a single pathogen [5]–[9] and for serological assays against several mixed pathogens [10], [11]. If the systems were optimized to couple microspheres to antigens of selected NTDs in sub-Saharan Africa, the prevalence survey or routine surveillance of NTDs could be managed easily, effectively, and in a timely manner.

We have been developing a new assay to apply this technology as part of a large-scale surveillance program to detect several infectious agents simultaneously and efficiently. As we report here, we evaluated the developed assay system, and used the system to survey seroprevalence in Kenya. We go on to discuss the possibility of large-scale comprehensive surveillance programs for NTDs and other infectious diseases in Africa.

## Methods

### Determination of antigen structures and antigen preparations

We chose cholera toxin (CTX), as well as 7 recombinant antigens, to measure IgG antibodies to each antigen assayed in this study (Table 1). CTX was obtained as purified native *Vibrio cholerae* CTX subunit A plus B purchased from Sigma-Aldrich (#C8052, MO, USA). The recombinant antigens were as follows: C-terminal part of the *Entamoeba histolytica* intermediate subunit (C-IgL) of galactose- and N-acetyl-d-galactosamine-inhibitable lectin for amebiasis [12]; *Leishmaniasis donovani* kinesin-related protein KRP42 for visceral leishmaniasis [13]; *Toxoplasma gondii* surface antigen 1 (SAG1) for toxoplasmosis [14]; *Wuchereria bancrofti* SXP1 for lymphatic filariasis [15]; and *HIV1* gag(MA+CA), gp41 ectodomain, and gp120 for HIV [16].

**Table 1:** Structure of recombinant antigens.

Pathogen	Antigen	Vector	Fusion tag on N-terminus	Antigen Region	Fusion tag on C-terminus	GenBank #
*Entamoeba histolytica*	C-IgL	pET19b	MGHHHHHHHHHHSSGHIDDDDKHMLE	603–1088		AF337950
*Leishmania donovani*	KRP42	pET52b	MASWSHPQFEKGALEVLFQGPGYQDP	1–337	IEFHHHHHHVDAAAELALVPRGSSAHHHHHHHHHH	BAF34578
*Toxoplasma gondii*	SAG1	pET52b	MASWSHPQFEKGALEVLFQGPGYQDP	61–300	LEVDAAAELALVPRGSSAHHHHHHHHHH	AAO61460
*Wuchereria bancrofti*	SXP1	pET52b	MASWSHPQFEKGALEVLFQGPGYQDPVTSSLNLTK	1–153	IEFHHHHHHLQVDAAAELALVPRGSSAHHHHHHHHHH	AAC17637
*Human Immunodeficiency Virus type 1*	gag	pET52b	MASWSHPQFEKGALEVLFQGPGYQDP	1–363	VDAAAELALVPRGSSAHHHHHHHHHH	AAB50258
	gp120	pET52b	MASWSHPQFEKGALEVLFQGPGYQDP	34–511	VDAAAELALVPRGSSAHHHHHHHHHH	AAB50262
	gp41	pET52b	MASWSHPQFEKGALEVLFQGPGYQ	512–683	ELALVPRGSSAHHHHHHHHHH	AAB50262
*Mycobacterium tuberculosis*	CFP10	pET52b	MASWSHPQFEKGALEVLFQGPGYQDP	1–100	AAAELALVPRGSSAHHHHHHHHHH	ZP_04982462
	ESAT6	pET52b	MASWSHPQFEKGALEVLFQGPGYQDP	1–95	AAAELALVPRGSSAHHHHHHHHHH	NP_338543

Note: For *Vibrio cholerae*, cholera toxin subunit A and B, which were not recombinant protein, were used in this study.

Antigens of *Mycobacterium tuberculosis*; CFP10 and ESAT6 [17], were part of the initial panel in the simultaneous microsphere-based multiplex assays. The descriptions for these antigens are retained in the methods section; however, they are omitted from the results and discussion due to poor the sensitivity.

The recombinant antigens were expressed in *Escherichia coli*. DNA encoding the recombinant antigens were amplified by polymerase chain reaction (PCR) and cloned into pET52b vectors or (for the C-IgL-encoding fragment only) pET19b. The resulting expression vectors encoded the respective epitopes as antigenic regions with fusion tags. Accession numbers for the respective antigens are provided in Table 1. The construction of the C-IgL expression vector has been described previously [12].

### Antigen purification

Each expression plasmid was transformed into BL21Star (DE3) pLysS chemically competent cells (Invitrogen, Carlsbad, CA, USA) and grown in LB broth containing 50 µg/ml carbenicillin and 34 µg/ml chloramphenicol. At an approximate density of 1.0 A_600_, protein expression was induced with 1 mM IPTG for 2–3 h at 37°C. After harvesting cells by centrifugation, the pellet was suspended in BugBuster (MERCK-Millipore, Darmstadt, Germany) containing 25 unit/ml Benzonase Nuclease. Protein solubilization and nucleotide degradation was performed in a rotating tube at room temperature for 20 min. Soluble and insoluble materials were separated by centrifugation at 14000×*g* at 4°C for 10 min.

*HIV1* gag, KRP42, and CFP10 were purified from soluble fractions by two-step affinity purification on COSMOGEL His-Accept resin (NACALAI TESQUE, Kyoto, Japan) and Strep-Tactin Superflow Plus resin (QIAGEN, Hilden, Germany) according to each manufacturer's instructions, with modifications as follows. In brief, the soluble fraction was loaded on His-Accept resin equilibrated with BugBuster, then washed once with BugBuster. After further washing twice with wash buffer (50 mM phosphate buffer, 0.5 M NaCl, 0.01% Tween 20, pH 8.0), antigen was eluted with elution buffer (50 mM phosphate buffer, 0.5 M NaCl, 0.01% Tween 20, 500 mM imidazole, pH 8.0). The eluate was diluted twice with wash buffer to reduce the imidazole concentration, then directly applied to Strep-Tactin resin. After washing the resin twice with wash buffer (100 mM Tris-HCl, 150 mM NaCl, 1 mM EDTA, pH 8.0), the antigen was eluted with elution buffer (100 mM Tris-HCl, 150 mM NaCl, 1 mM EDTA, 2.5 mM desthiobiotin, pH 8.0). The purified antigen then was dialyzed in phosphate-buffered saline (PBS) without calcium and magnesium[PBS(-)]. *HIV1* gp120, *HIV1* gp41 ectodomain, IgL, SAG1, SXP1, and ESAT6 were purified from the insoluble fractions. Preparation of inclusion bodies was performed with BugBuster protein extraction reagent according to the protocol for the protein refolding kit (TB234 12/98, Novagen, Inc, WI, USA). The prepared inclusion bodies (which primarily contained expressed antigens) were suspended in 0.3% N-lauroylsarcosine in CAPS buffer (pH 11), and then rotated for 15 min at room temperature. Insoluble materials were removed by centrifugation at 14000×*g* for 10 min at 4°C. Solubilized samples were dialyzed in 0.3% N-lauroylsarcosine/PBS(−) and purified by His-Accept resin as described previously, with the modification that His-Accept resin was equilibrated with 0.3% N-lauroylsarcosine/PBS(−) instead of BugBuster. Purified antigens were dialyzed in 0.3% N-lauroylsarcosine/PBS(−).

The protein concentrations of soluble antigens were determined with a Quick Start Bradford Protein Assay Kit (Bio-Rad Laboratories, CA, USA) and concentrations of insoluble antigens were determined using a Pierce 660-nm Protein Assay Kit with Ionic Detergent Compatibility Reagent (Thermo Scientific, Rockford, IL, USA).

Each of the purified proteins (5.0 µg/lane) was separated on NuPAGE Novex 4%–12% Bis-Tris Gel in 1× MES SDS Running Buffer (Life Technologies Corporation, Carlsbad, CA, USA) under reducing conditions. The gel was stained with Coomassie Brilliant Blue.

DNA and protein data were analyzed to predict the molecular weights of antigens by CLC Main Workbench 6 software (CLC bio, Aarhus, Denmark). Antigen homologies also were analyzed with the National Center for Biotechnology Information (NCBI) Protein Basic Local Alignment Search Tool (BLAST).

### Coupling antigens with microspheres

Following purification, the individual antigens were coupled with microspheres (MagPlex) using a separate color for each antigen. Two types of microspheres made from different materials are commercially available from Luminex Corporation: MicroPlex microspheres and MagPlex microspheres (Luminex Corporation, Austin, TX, USA). A high nonspecific immunological background of MicroPlex microspheres in serological assays has been reported [18], [19]. For immunoreactions, filter-bottom plates (MultiScreenHTS -BV, MERCK-Millipore) washed using an ELx405 microplate washer with a vacuum system were used for Microplex, and Bio-Plex Pro flat-bottom plates (Bio-Rad) and an ELx405 microplate washer (BioTek, Winooski, VT, USA) with magnetic plates were used for MagPlex. After comparing two types of non-treated microspheres for nonspecific immunological background in a single-plex format for 148 serum samples collected in the field survey in Kenya, MagPlex microspheres were chosen for our assay development.

Anti-human IgG antibody and CTX were dialyzed in PBS (−) before the coupling reaction.

Each antigen, the anti-human IgG antibody, and CTX were coupled with a different “color” of MagPlex microspheres in a one-to-one pairing following the manufacturer's instructions, with modifications as follows. In brief, carboxyl groups on the microspheres were activated with EDAC (1-ethyl-3-[3-dimethylaminopropyl] carbodiimide hydrochloride, Thermo Scientific Inc.) and S-NHS (N-hydroxysulfosuccinimide, Thermo Scientific Inc.) in activation buffer (0.1 M NaH_2_PO_4_, pH 6.2) for 30 min at room temperature. After incubation, the microspheres were washed with PBS (−), pH 7.4. Antigen then was coupled to the microspheres for 2 hours at room temperature with gentle agitation. Antigen amounts in the coupling reaction for 1.25 million microspheres were determined after a titration experiment, and were as follows: 3 µg of KRP42; 10 µg of gag; cholera toxin, and 30 µg of C-IgL, SAG1, CFP10, and ESAT6. After reaction, the coupled microspheres were washed with PBS (−), then free carboxyl groups were blocked by 50 mM ethanolamine pH 8.5 (Wako, Osaka, Japan) for 30 min at room temperature. The microspheres were washed twice with StabilGuard (SurModics, Eden Prairie, MN, USA), and the concentration was adjusted to 1000 microspheres/µl in StabilGuard and stored at 4°C. To determine serum reaction during the assay, 100 µg of mouse monoclonal antibody raised against a human IgG Fab fragment (Clone 4A11, EXBIO, Vestec, Czech Republic) was coupled to microspheres and served as a positive control.

### Multiplex assays and evaluations

Two microliters of serum was diluted with 98.0 µl of staining buffer [0.1% bovine serum albumin, 0.05% Tween20, 0.05% sodium azide in PBS (−), pH 7.5]. In total, 16 different-colored microspheres were used for the assays, as follows: 15 colors corresponding to 14 antigens (6 antigens were not included in this paper) and one anti-human IgG antibody and one color corresponding to non-coupled microspheres (used to monitor the level of nonspecific reactions with the microspheres). Suspensions (1.0 µl at 1000 microspheres/µl) of each microsphere species were added sequentially to 84 µl of staining buffer. The resulting 100-µl suspensions, which included 15 different types of coupled microspheres and a non-coupled microsphere, were added to the wells containing the diluted serum.

A binding reaction was performed for 30 min at room temperature while shaking at 750 rpm in the dark. The plate then was transferred to an ELx405 microplate washer (BioTek, Winooski, VT, USA) with a magnetic plate for three washing steps with washing buffer [0.05% Tween 20, 0.05% sodium azide in PBS(−), pH 7.5]. While the binding reactions were in progress, phycoerythrin-conjugated goat anti-human IgG F(c) F(ab')_2_ fragment (detection antibody; #709-1817, Rockland Inc., Gilbertsville, PA, USA) was diluted to a phycobiliprotein concentration of 2.0 µg/ml in staining buffer. The diluted detection antibody was added to the wells and incubated for 30 min at room temperature with shaking at 750 rpm in the dark. After incubation, the wells were washed as described previously. The microspheres were suspended on a shaker at 750 rpm for 5 min after addition of 125 µl of wash buffer to each well. Fluorescence was monitored using a Bio-Plex200 system (Luminex Corporation, Austin, TX, USA).

### Stability of the antigen-coupled microspheres during storage

Following coupling, microspheres were stored at 4°C; all assays were conducted within 26 days of the coupling reaction. The stability of the coupled microspheres was confirmed by combining coupled microspheres with a mixed positive control serum (in triplicate) for all antigens and monitoring the assay values for all antigens regularly (approximately 4–5 times/week) over 26 days.

### Evaluation of the antigen-coupled microspheres using sera of patients and healthy Japanese

The evaluation of the microsphere-based multiplex assay system itself was performed using sera from infected patients (as positive controls) and sera from healthy Japanese (as negative controls). The immune status of the positive control sera was confirmed by standardized methods or routine clinical processes for the target infection and not clinically confirmed for more than two simultaneous infections. For HIV, sera were collected at the Kitale District Hospital, Saboti County, Kenya. These sera were screened using the following diagnostic kits; Determine HIV 1/2 tests (Abbott Diagnostic Division, Hoofddorp, Netherlands) and Uni-Gold HIV Kits (Trinity Biotech, Bray, Ireland). Positive control sera for cholera were collected from the Machakos Provincial General Hospital, Machakos County, Kenya. For *V. cholerae*, sera were obtained from a routine cholera surveillance program in Kenya; all were confirmed by PCR using isolates obtained following selection from patient stool specimens using TCBS medium. For *T. gondii*, we had difficulty finding symptomatic toxoplasmosis patients in a hospital setting. Therefore, we used sera obtained as part of the field survey used in this study; these sera were used after confirmation of *Toxoplasma* status by RDT Kits (OnSite Toxo IgG/IgM Rapid Test-Cassette, CTK Biotech, CA, USA). For *W. bancrofti*, sera were obtained from Kenya Medical Research Institute, Kenya; diagnoses were confirmed parasitologically by identifying microfilariae in blood smears drawn at night. For *E. histolytica*, sera were obtained from the International Center for Diarrheal Disease Research, Bangladesh; diagnoses were confirmed by detection of *E. histolytica*-specific DNA in liver abscess pus specimens. For *L. donovani*, sera were obtained from the Rajshahi Medical College in Bangladesh; diagnoses were parasitologically confirmed by microscopic examination of spleen aspirates. As negative controls, serum samples from 40 healthy Japanese individuals were used to calculate cut-off values for each antigen for the purpose of evaluation; seronegativity of these samples was assumed based on the rarity of infection in Japan for the pathogens examined for this study; infections by HIV, *W. bancrofti*, *E. histolytica*, *V. cholerae* cases are quite rare and *L. donovani* infection has not been reported according to the National surveillance in Japan and other research reports [20]–[23]. The seropositivity of *T. gondii* is reported as 10.3% in pregnant women in Japan [24]. The cut-off values for the evaluation were calculated as the means plus three standard deviations (SD) of the distributions of median fluorescence intensity (MFI) values from two independent assays. The details of the number of positive and negative control sera are provided in Table 2.

**Table 2:** Summary of serum reactivity from positive and negative controls.

Pathogen	Antigen	Cut-off value	Mean of MFI*	S.D.	Positive control			Negative control			Sensitivity		Specificity	
					Total	Positives	False negatives	Total	Positives	False positives	%	95%CI	%	95%CI
HIV1	gp41	171.16	104.48	22.23	50	50	0	40	0	0	100.0%	(92.9–100)	100.0%	(91.2–100)
	gag	193.66	95.15	32.84	50	40	10	40	2	2	80.0%	(66.3–90)	95.0%	(83.1–99.4)
	gp120	213.34	133.75	26.53	50	40	10	40	0	0	80.0%	(66.3–90)	100.0%	(91.2–100)
*V. cholerae*	CTX	224.51	95.5	43	7	5	2	40	1	1	71.4%	(29–96.3)	97.5%	(86.8–99.9)
*E. histolytica*	C-IgL	144.22	83.43	20.27	20	20	0	40	0	0	100.0%	(83.2–100)	100.0%	(91.2–100)
*L. donovani*	KRP42	277.12	87.48	63.21	16	15	1	40	1	1	93.8%	(69.8–99.8)	97.5%	(86.8–99.9)
*W. bancrofti*	SXP1	485.07	176.68	102.8	20	19	1	40	1	1	95.0%	(75.1–99.9)	97.5%	(86.8–99.9)
*T. gondii*	SAG1	435.15	91.48	114.56	19	18	1	40	2	2	94.7%	(74–99.9)	95.0%	(83.1–99.4)

*Means of the distributions of median fluorescence intensity (MFI) among 40 negative control serum samples measured by the multiplex system

### Population-based serological survey: Study sites and selection of individuals for blood sampling

For the serological survey, a database corresponding to two Health and Demographic Surveillance System (HDSS) sites managed by the Institute of Tropical Medicine, Nagasaki University, and the Kenya Medical Research Institute (KEMRI) was used for blood sampling. As of 2011, we have established and implemented two HDSS programs in the western (Mbita site) and coastal (Kwale site) areas of Kenya, where there are distinct disease burdens and cultures as described in detail elsewhere [25]. Of 77,887 individuals recorded in the HDSS database, 4,600 individuals were randomly selected by HDSS site, sex, and age group. A total of 10 age groups were divided by five-year intervals up to the age of 45 years; individuals older than 45 years of age were consolidated as a group. As a result, all individuals were categorized into 40 groups, and 115 individuals were randomly selected from each group anticipating a 15% loss of participation.

### Blood sampling

Between July 2011 and August 2011, a blood sampling survey was conducted at the Kwale and Mbita HDSS sites according to the list of 4,600 randomly selected individuals. Each site was divided into 20 wards according to the HDSS address system. Blood samples were collected in each ward on a separate day. On the day prior to blood sampling, village volunteers visited the selected households, explained the purpose of the study, and invited individuals to the place where blood sampling would be performed. Blood sampling sites typically consisted of elementary schools and health centers. For those who did not come to the sampling place, village volunteers took the selected individuals to the site using project vehicles. For those who were not available on the scheduled day, additional days for blood sampling were scheduled. After re-explanation of the study and obtaining informed consent (confirmed by participant signature), blood samples were drawn using 3-ml sampling tubes for those who were more than five years of age and 0.5-ml tubes for children under 5 years of age. For children less than 18 years of age, the purpose of the study was explained to a parent or a guardian and a written agreement to participate was obtained. Following collection, blood samples were stored in a refrigerator and then sent to the main laboratory of the project (in Nairobi) by shipping in a mobile refrigerator or courier package service with dry ice. Microsphere-based multi-serological assays for all samples were conducted in the laboratory.

### Mathematical models for cut-off values

High immunological backgrounds have been reported among African populations [26], [27]; therefore, the cut-off values defined by Japanese serum samples could have been too sensitive, which would lead to false positives. Additionally, from public health point of view, populations currently infected (high antibody titer) and those infected in the past but treated (low or intermediate antigen titer) must be differentiated. For this purpose, the cut-off values using Japanese or negative control sera would not be suitable.

To optimize cut-off values for the study sample population, we applied the concept of finite mixture distribution, which is used to model data from populations known or suspected to contain hidden separated subpopulations [28]. We assumed that this population could be separated into several subpopulations with regard to immune status. For example, a population might be split into “immunity,” “modified immunity,” and “without immunity” categories if one assumed that there were three hidden subpopulations; and in some infectious agents, it might be separated into more than three immune statuses [29]. The finite mixture model has been used in past studies to optimize cut-off points from survey data from populations for several infectious diseases [29], [30]. In the present study, a distribution of all assay values (MIF values) for each antigen was separated into components using the “fmm” (finite mixture model) command [31] of Stata statistical software (version 12.1; Stata Corporation, TX, USA). To select the best model, which also returns the component number, we applied the theory of Bayesian Information Criterion (BIC). To build the model, individual data were weighted according to the proportions of site, sex, and age group that were obtained from the population data of HDSS. After separating the distribution of assay values, a cut-off point for each antigen was optimized as the index maximum value [32], which was calculated as sensitivity-(1-specificity) obtained from the distribution separated by a finite mixture model. Sensitivity and specificity were defined as a proportion of two distributions beyond the Youden index value. For the antigens in which values were separated into four or five normal distributions, two cut-off values were set for evaluation of seroprevalence: one cut-off value with high sensitivity, and a second with high specificity. For HIV, we defined seropositivity as two or more positives among the three antigens tested.

### Ethical considerations

The survey protocol was approved by the Ethical Review Committee of Kenya Medical Research Institute (KEMRI SSC No. 1934) and the Ethical Committee of the Institute of Tropical Medicine, Nagasaki University (10061550 and 10122261-2). Prior to the survey, we sensitized the communities in the HDSS sites by inviting village elders and community members to learn about the project. For blood collection, we explained the project in advance and collected blood samples from those who agreed to participate and signed consent forms. For children <18 years of age, we explained the project to their parent or guardian and collected blood samples after obtaining the parent/guardian agreement and signature. This procedure (to obtain consent from the parent or guardian of children <18 years of age) was approved by the both ethical committees of Kenya Medical Research Institute and the Institute of Tropical Medicine, Nagasaki University.

### Accession numbers/ID numbers for genes and proteins

GenBank accession numbers for each antigen were as follows; *Entamoeba histolytica* (C-IgL): AF337950, *Leishmania donovani* (KRP42): BAF34578, *Toxoplasma gondii* (SAG1): AAO61460, *Wuchereria bancrofti* (SXP1): AAC17637, Human Immunodeficiency Virus type 1 (gag): AAB50258, Human Immunodeficiency Virus type 1 (gp120): AAB50262, Human Immunodeficiency Virus type 1 (gp41): AAB50262, *Mycobacterium tuberculosis* (CFP10): ZP_04982462, and *Mycobacterium tuberculosis* (ESAT6): NP_338543.

## Results

### Evaluation of antigen purification

Proteins prepared for coupling on microspheres were analyzed by sodium dodecyl sulfate polyacrylamide gel electrophoresis (SDS-PAGE). All antigens were of good purity as shown in Figure S1. All recombinant antigens except gp41 ectodomain were detected at the predicted molecular weight calculated based on their amino acid sequences. Regarding gp41 ectodomain, the predicted molecular weight was 24.9 kDa; however, the expressed protein ran as two major bands of approximately 24 kDa and 48 kDa. The bigger size band was presumed to be the dimer of the recombinant antigen. For CTX, two major bands were detected, consistent with the expected presence of a single A-subunit (approximately 27.2 kDa) and five B-subunits (approximately 11.6 kDa) in each CTX molecule. Two bands also were detected in the anti-human IgG antibody, consistent with the expected tertiary structure of light chains (approximately 25 kDa) and heavy chains (approximately 50 kDa).

### Evaluation of the microsphere-based multiplex assay

**Figure 1:**
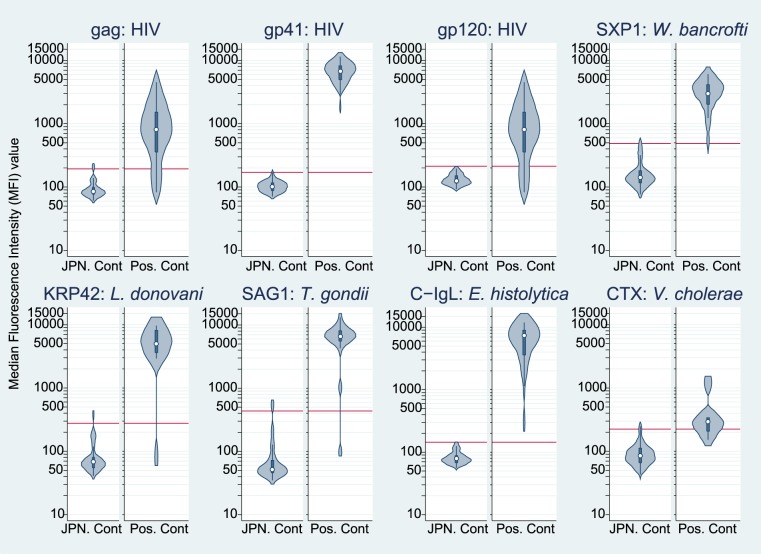
Serum reactivity by the multiplex assays for 8 antigens among healthy Japanese and clinically diagnosed positive sera: HIV (gag, gp41and gp120), *W. bancrofti* (SXP1), *L. donovani* (KRP42), *T. gondii* (SAG1), *E. histolytica* (C-IgL), and *V. cholerae* (CTX) antigens. Red horizontal lines represent cut-off values calculated as the means plus three standard deviations (SDs) of the distributions of median fluorescence intensity (MFI) values of 40 healthy Japanese serum samples.

The ranges of MFI values for each antigen are shown in Figure 1 for negative and positive controls (separately) using a violin plot format that shows the median, a box indicating the interquartile range, spikes extending to the upper- and lower-adjacent values, and kernel density estimation. Means, SDs, and calculated cut-off values for each antigen for validation are shown in Table 2 along with seropositive numbers for positive and Japanese controls. The cut-off values varied by antigen, ranging from 144.2 for C-IgL to 485.1 for SXP1. Sensitivities and specificities (respectively) for each antigen were as follows: 100.0% (95%CI: 92.9–100.0) and 100.0% (95%CI: 91.2–100.0) for gp41 (HIV1), 80.0% (95%CI: 66.3–90.0) and 95.0% (95%CI: 83.1–99.4) for gag (HIV1), 80.0% (95%CI: 66.3–90.0) and 100.0% (95%CI: 91.2–100.0) for gp120 (HIV1), 71.4% (95%CI: 29.0–96.3) and 97.5% (95%CI: 86.8–99.9) for CTX (*V. cholerae*), 100.0% (95%CI: 83.2–100.0) and 100.0% (95%CI: 91.2–100.0) for C–IgL (*E. histolytica*), 93.8% (95%CI:69.8–99.8) and 97.5 (95%CI: 86.8–99.9) for KRP42 (*L. donovani*), 95.0% (95%CI: 75.1–99.9) and 97.5 (95%CI: 86.8–99.9) for SXP1 (*W. bancrofti*), 94.7% (95%CI: 74.0–99.9) and 95.0% (95%CI: 83.1–99.4) for SAG1 (*T. gondii*).

### Temporal stability of stored microspheres coupled with antigens

The MFI values of mixed positive sera were measured in the assay processes. The period ranged from one storage day after the microspheres were coupled with antigens to 26 storage days. The assays were not conducted every day, so that the measurement of MFI of the mixed positive serum was not performed on a daily basis. The coefficients of variation of the MFI measurements were <13%, indicating that the assay should be stable for at least 26 days (Figure S2).

### Population-based serological survey

**Figure 2:**
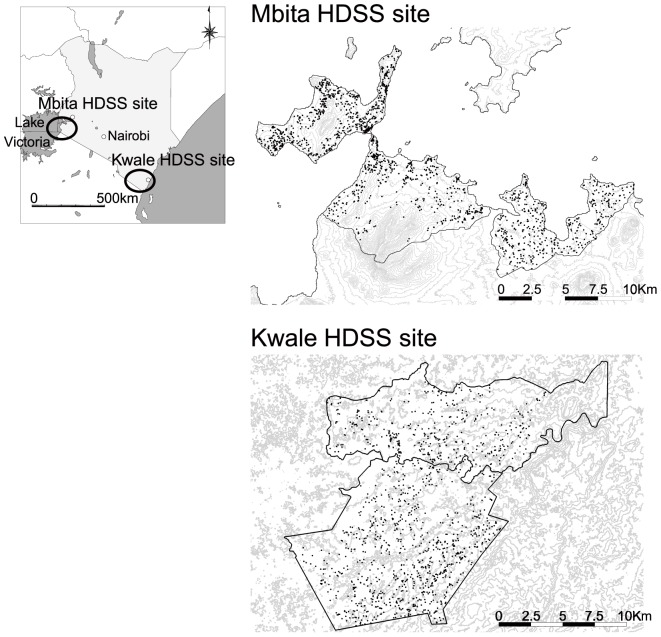
Geographic distribution of sampled populations from the two HDSS sites. Each dot represents individuals selected for the survey. From each HDSS site, 2200 residents were selected by sex and age group. At the Kwale HDSS site, 1453 individuals agreed and participated in blood sample collection. At the Mbita HDSS site, 1958 individuals agreed and participated.

A total of 3411 individuals agreed to blood collection, including 1453 individuals from the Kwale HDSS and 1958 individuals from the Mbita HDSS. The geographical distributions of the sampled populations are shown in Figure 2, and sex and age group distributions of the participating individuals are shown in Table 3. Participation proportions from the two sites were 63.2% (1,453/2,300) from Kwale and 85.1% (1,958/2,300) from Mbita.

**Table 3:** Age and sex distribution of serological survey participants at the two HDSS sites.

years of age	Kwale site							
	Females			Males			Total	
	Sample No.	Population	Weight*	Sample No.	Population	Weight*	Sample No.	Population
0–4	91	2707	30	89	2910	33	180	5617
5–9	82	2874	35	88	3012	34	170	5886
10–14	88	2565	29	84	2835	34	172	5400
15–19	66	1969	30	76	2166	29	142	4135
20–24	58	1685	29	56	1373	25	114	3058
25–29	59	1320	22	55	1078	20	114	2398
30–34	77	1109	14	52	866	17	129	1975
35–39	79	777	10	55	645	12	134	1422
40–44	79	651	8	67	545	8	146	1196
≥45	81	2179	27	71	2004	28	152	4183
Total	760	17836		693	17434		1453	35270

years of age	Mbita site							
	Females			Males			Total	
	Sample No.	Population	Weight*	Sample No.	Population	Weight*	Sample No.	Population
0–4	102	3343	33	97	3353	35	199	6696
5–9	106	3763	36	107	3806	36	213	7569
10–14	105	3108	30	105	3105	30	210	6213
15–19	99	1998	20	101	2137	21	200	4135
20–24	86	2186	25	85	1690	20	171	3876
25–29	100	1983	20	93	1643	18	193	3626
30–34	97	1335	14	92	1455	16	189	2790
35–39	102	871	9	87	870	10	189	1741
40–44	103	723	7	91	539	6	194	1262
≥45	108	2911	27	92	1798	20	200	4709
Total	1008	22221		950	20396		1958	42617

years of age	Total							
	Females			Males			Total	
	Sample No.	Population		Sample No.	Population		Sample No.	Population
0–4	193	6050		186	6263		379	12313
5–9	188	6637		195	6818		383	13455
10–14	193	5673		189	5940		382	11613
15–19	165	3967		177	4303		342	8270
20–24	144	3871		141	3063		285	6934
25–29	159	3303		148	2721		307	6024
30–34	174	2444		144	2321		318	4765
35–39	181	1648		142	1515		323	3163
40–44	182	1374		158	1084		340	2458
≥45	189	5090		163	3802		352	8892
Total	1768	40057		1643	37830		3411	77887

*Weights represent the probability that an individual was selected into the sample from a population. The weights are calculated by taking the inverse of the sampling fraction; and used for finite mixture models.

**Figure 3:**
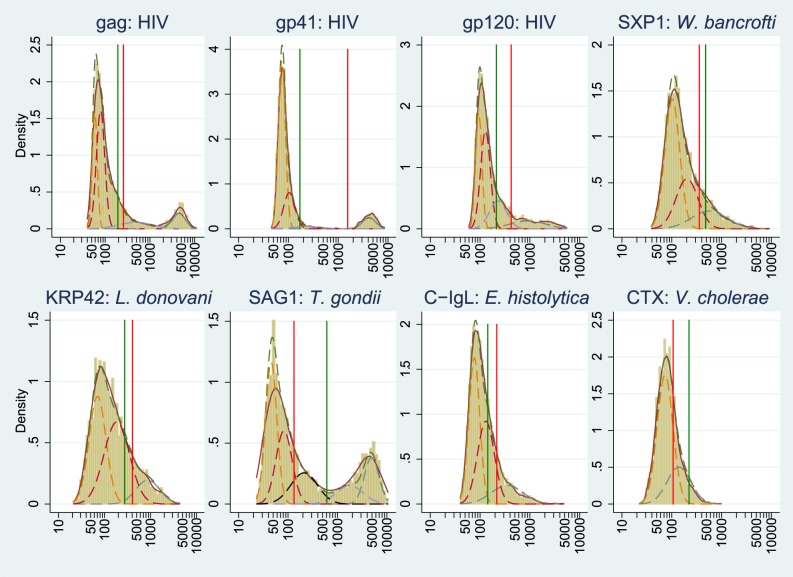
Distribution of median fluorescence intensity (MFI) for antigens of HIV (gag, gp41, and gp120), *W. bancrofti* (SXP1), *L. donovani* (KRP42), *T. gondii* (SAG1), *E. histolytica* (C-IgL), and *V. cholerae* (CTX). Solid lines: Kernel density of the distribution. Actual distributions are expressed as histograms. Dotted lines: Normal distribution separated by mathematical models (finite mixture models) used to calculate cut-off values for each antigen. Vertical lines: Green represents cut-off values calculated using Japanese volunteers; red represents those calculated by two normal distributions obtained by mathematical models. MFI: outcome value of the microsphere-based assay system; these values are roughly equivalent to antibody titer.

The distributions of immunoassay values for each antigen are shown in Figure 3 along with separated subpopulations obtained by the finite mixture model; cut-off values were obtained from the distribution of assay values among Japanese populations as well as from the Youden index calculation. According to finite mixture models, the distribution of immunoassay values (MFI values) were separated into two to five components, with specific numbers as follows: HIV (gp41), four; HIV (gp120), four; HIV (gag), five*; W. bancrofti* (SXP1), three; *L. donovani* (KRP42), three; *E. histolytica* (C-IgL), three; cholera toxin (CTX), two; *T. gondii* (SAG1), five. For each antigen, the model with the lowest BIC value was selected. The BIC results are shown in Table S1.

From the distributions separated by the model, optimized cut-off values for the immunoassays were calculated. For some antigens, the distributions were separated into four or five components; two cut-off value points were calculated to compare the differences of the cut-off values, although the third and fourth distributions were used for HIV because there were apparent separations between those distributions. Using the two separated distributions, we optimized cut-off values for each antigen by calculating the Youden index. The optimized cut-off value was defined as the maximum value of the Youden index for each antigen. The distributions of antibody MFI values to *T. gondii* were separated into four or five normal distributions. For *T. gondii*, only the cut-off value with the high-specificity setting was used because of the separable shape of the distribution. Sensitivities and specificities were obtained using the above-calculated cut-off values (using the same positive and negative control sera as used for the validation process); the results are shown in Table S2.

In Table 4, seropositivities simultaneously measured by the microsphere-based multi-serological assay are shown according to pathogens from the Kwale and Mbita sites, respectively. Seropositivities of each pathogen for the whole population were as follows for the Kwale and Mbita HDSS sites (respectively): HIV, 3.0% and 20.1%; *L. donovani*, 12.6% and 17.3%; *E. histolytica*, 12.8% and 16.6%; *T. gondii*, 30.9% and 28.2%. Seropositivities for *W. bancrofti* were 21.8% and 13.5% and those for *V. cholerae* were 31.7% and 24.9%. These results might be carefully interpreted taking immunological cross reactions into account between *W.* bancrofti-SXP1 antigen and other parasitic infection; and between cholera toxin and enterotoxigenic *E. coli* (ETEC). The values of seropositivities by sex, age, and site are shown in Table S3 and S4.

**Table 4:** Sero-positive proportions measured by multiplex assays and prevalence ratio at the two study sites.

	Female		Male		Total		Prevalence Ratio	
Pathogen	Positive	95%CI	Positive	95%CI	Positive	95%CI	Ratio	95%CI
1) Kwale site								
HIV	3.70%	(2.3–5.0%)	2.30%	(1.2–3.4%)	3.00%	(2.1–3.9%)	ref	
*W. bancrofti*	19.20%	(16.4–22.0%)	24.70%	(21.5–27.9%)	21.80%	(19.7–23.9%)		
*L. donovani*	11.10%	(8.8–13.3%)	14.30%	(11.7–16.9%)	12.60%	(10.9–14.3%)		
*E. histolytica*	12.50%	(10.1–14.9%)	13.10%	(10.6–15.6%)	12.80%	(11.1–14.5%)		
*V. Cholerae*	29.60%	(26.4–32.9%)	33.90%	(30.4–37.4%)	31.70%	(29.3–34.1%)		
*T. gondii*	31.80%	(28.5–35.2%)	29.90%	(26.5–33.3%)	30.90%	(28.5–33.3%)		
2) Mbita site								
HIV	24.10%	(21.5–26.7%)	15.80%	(13.5–18.1%)	20.10%	(18.3–21.8%)	6.63	(6.32–6.93)
*W. bancrofti*	12.60%	(10.6–14.6%)	14.50%	(12.3–16.8%)	13.50%	(12.0–15.0%)	0.62	(0.47–0.77)
*L. donovani*	17.30%	(14.9–19.6%)	17.40%	(15.0–19.8%)	17.30%	(15.6–19.0%)	1.37	(1.21–1.54)
*E. histolytica*	17.30%	(14.9–19.6%)	15.90%	(13.6–18.2%)	16.60%	(15.0–18.2%)	1.3	(1.13–1.46)
*V. Cholerae*	24.20%	(21.6–26.9%)	25.70%	(22.9–28.5%)	24.90%	(23.0–26.8%)	0.79	(0.68–0.90)
*T. gondii*	31.30%	(28.5–34.2%)	24.90%	(22.2–27.7%)	28.20%	(26.2–30.2%)	0.91	(0.81–1.02)

## Discussion

The aim of this study was to develop and evaluate a microsphere-based multiplexed serological assay for infectious diseases that makes it possible to conduct immunological assay for several NTDs concurrently. This assay system enabled us to determine the epidemiological and geographical distributions of these diseases,and to monitor the effects of control programs of NTDs and other infectious diseases, particularly those with similar geographical patterns [4]. For development of the system, we applied IgG antibody measurement. Such antibody titering is generally considered unsuitable for clinical diagnosis of infectious diseases, because the technique does not permit distinguishing between present and past infections. However, from an epidemiological point of view, IgG antibody prevalence to target antigens is effective for long-term monitoring and surveillance for chronic infectious diseases like NTDs in communities [4].

During the development of the assay system, two major issues had to be addressed prior to applying this system for our field survey. The first issue was antigen production. Antigens suitable for antibody detection had to be selected and prepared by coupling with microspheres. In the development process, we focused primarily on recombinant antigens; recombinant antigens were expected to provide higher specificity than crude antigens, which were expected to induce non-specific antibody reactions in serum. Purity of recombinant antigens is essential to avoid unintended binding of antibodies to non-specific bacterial antigens. We purified soluble antigens (derived by expression from the pET52b or pET19b expression vector) by two-step affinity chromatography using N-terminal StrepTag II- and polyhistidine-tag. In our hands, this two-step process was effective for obtaining highly purified antigens, particularly for antigens with low expression levels.

The second issue was that of cut-off value evaluations. We tried to set cut-off values for each antigen based on assay value (MFI value) distribution among infection-negative groups (specifically, in the sera obtained from a Japanese population). However, a high immunological background had been reported in African populations [26], [27]. As the result, cut-offs determined using the serum of a Japanese population or from developed countries may correspond to values lower than those expected in infected populations, leading to elevated frequencies of false positives in an African population.

Additionally, the sampled population might have different immune status groups; for example, populations currently infected (high antibody titer); those infected in the past but subsequently treated or lacking active infection (low or middle antigen titer); and those never infected (lower antibody titer). For public health intervention to control infectious diseases, the surveillance should incorporate the currently infected population at the community level. To avoid such false results and to determine the mixed distribution of different immune statuses among sampled population, we applied finite mixture models to identify hidden population groups in the distribution of assay values within the sampled population [29], [30].

Distinct immune distributions in the assay values can be identified by including different geographical areas with distinct endemic for each pathogen. Ideally, if we could cover three geographical areas with different endemicity (e.g., non-endemic areas, middle endemic areas, and highly endemic areas), the ideal cut-off values for each pathogen would be obtained. We covered two distinct areas with different endemicity of infectious diseases in Kenya for this study, although endemicity did not differ for all of the examined pathogens.

Regarding interpretation of the results from the microsphere-based multiplex serological assays, the theoretical principle behind the technology for multiplex assays is the same as that for ELISA; therefore, the interpretation for each assay is expected to be similar to that for the interpretation of ELISA results. The MFI distribution for HIV1 antigens was sufficient to permit distinguishing between samples that were negative and positive for infection, as mentioned in the supplementary text. In terms of geographical differences, seropositivities for HIV antigens in Mbita were higher than those in Kwale, both for males and females. Sorting by age group made the difference in seropositivities much clearer. Particularly in age groups older than the twenties, seropositivities rapidly increased up to approximately 50% in Mbita, but such trends were not observed in Kwale. This trend may reflect differences in HIV infection risk factors between the sites. Lower rates of seropositivity among the young population (<20 years old) may be attributed to the current situation of HIV control; however, results will need to be carefully monitored among all of these age groups.

Seropositivities for other pathogens can be interpreted in a fashion similar to that for HIV. Regarding toxoplasmosis, seropositivities to *T. gondii* increased linearly according to age both in Kwale and Mbita, meaning that exposure to *T. gondii* exists in both areas with similar probabilities for all age groups. Toxoplasmosis is a zoonotic parasitosis, and the risk of human infection in rural areas of Africa is common, since sheep and goats likely have been infected by *T. gondii*. Based upon our results, the risk of infection may be the same for all age groups, given that the prevalence of seropositivity increased linearly from the young age group to the elder age groups. *T. gondii* infection is reported as one of the risk factors for convulsive epilepsy in Africa [33]; thus, more active surveillance and control programs for *T. gondii* may be necessary for Africa women of child-bearing age.

**Figure 4:**
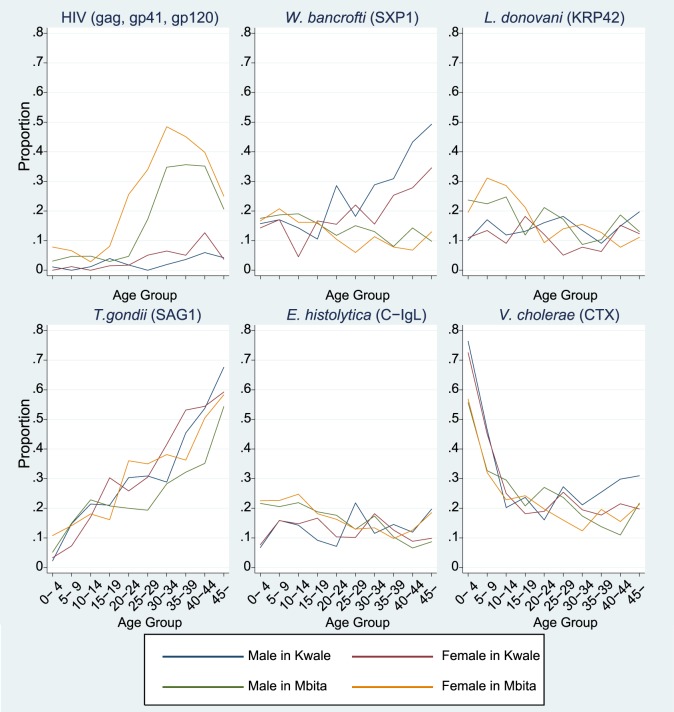
Age- and sex-specific prevalence of serological positives among sampled populations from Kwale and Mbita sites for HIV, *W. bancrofti*, *L. donovani, T. gondii*, *E. histolytica*, and *V. cholerae*. HIV positivity defined as at least two serological positives among three antigens tested (gag, gp41, and gp120).

Regarding the serological results for *L. donovani*, there was a report of an outbreak in northeastern Kenya in 2000 [34]. Although no similar report has been published regarding other regions, there may be undetectable sporadic outbreaks in other areas of Kenya; the seropositivity observed in the present study may reflect one or more such sporadic outbreaks. The seroprevalence was higher in the population <20 years old from the Mbita site (Figure 4). The cause for this higher positivity for *L. donovani* in the younger population of the Mbita site is unknown, although the trend might reflect cross-reactivity to other pathogens [35]. Further investigations are required to clarify this high frequency of seropositivity for *L. donovani*.

For the assay for *E. histolytica*, the recombinant surface antigen C-IgL was previously validated [12] and was used for seroepidemiology of *E. histolytica* in Chinese populations [36]. Seropositivities in all age groups were higher in our study areas compared with the Chinese population, in which seropositivities ranged from 3–8% [36]. Comparing the seropositivities among children from our two study sites, the rates for children from Mbita were generally higher than those for children from Kwale. This trend may be due to the difference in the source of drinking water and latrine distributions for both sites. In Mbita, most of the households use water directly fetched from Lake Victoria and there is low latrine usage; on the other hand, in Kwale, many households use water collected from water pipes and latrine distribution has improved in recent years [25].

For *W. bancrofti*, seropositivity should be interpreted carefully, though the seropositive proportion in Kwale was higher in those >20 years of age compared with the same cohort in Mbita and this result may reflect the past endemic status of filariasis and the effect of elimination programs for filariasis with mass drug administration (MDA) in recent years in the coastal area of Kenya, historically a region where filariasis was highly endemic [37], [38]. However, the seropositive rate in the young population in Mbita was approximately 10%, meaning that there might be some cross reactions between SXP1 and other parasitic diseases [39], because Mbita region is recognized as a non-endemic area of filariasis. Basically, for sero-diagnosis to SXP1 antigen, IgG4 subclass is used; however, our microsphere-based multiplex assay is to detect IgG antibody titers for multiple pathogens, simultaneously; therefore, our system might be capturing other cross-reactive IgG subclasses to other pathogens. To reduce the effect of the cross-reaction on prevalence estimation, further study must be done to add different type of antigens on our assay system.

Cholera seropositivity must be also interpreted with care, because cholera toxin and the enterotoxigenic *E. coli* (ETEC) are immunologically related and ETEC infection is common in developing countries [40]. Furthermore, in a case-control study on young child death in another area of Kenya, there was no case of cholera, though deaths due to ETEC were detected [41]. Therefore, the positive prevalence of cholera in the present study may reflect infection by ETEC in the study area. The decreasing trends of positivity are also consistent with the decreasing ETEC infection after five years of age in other countries [42]. Therefore, the detection of CTX by microsphere-based serological assay may not be practical.

Beyond the specific interpretations of the results of our study for individual pathogens, the largest advantage of the microsphere-based multi-serological assay is to facilitate comprehensive and simultaneous monitoring for multiple pathogens. Establishing seroepidemiological surveillance programs in different and larger regions is expected to provide a monitoring system to determine geographic and temporal trends for several infectious diseases. Such a multiplexed assay also is expected to permit the evaluation of control programs such as MDA, and to facilitate monitoring for re-emergence of infectious diseases of interest once it is decided that such diseases have been successfully controlled.

Selection of target pathogens and antigens are essential for any such surveillance program. The pathogens surveyed in this study were selected on a somewhat ad hoc basis, depending on antigen availability and comments from disease specialists. However, realistically pathogens must be selected according to the demand from the field and policy in the affected areas, as well as support from a large number of researchers working in the area. Further development would be enhanced by a platform to share information on pathogens; such a platform should be targeted and should incorporate antigens suitable for surveillance programs. By doing so, made-to-order surveillance programs suitable for specific areas with different situations and disease burdens can be devised. Furthermore, multiplexed assays should include several antigens for each single pathogen, to provide detection with high sensitivity and specificity. In Kenya, the national multi-year strategic plan for control of NTDs was published by the government in 2012 [43]. The target NTDs are schistosomiasis (bilharziasis), soil transmitted helminthiasis, lymphatic filariasis (elephantiasis), trachoma, leishmaniasis (Kala-azar), and hydatid disease (echinococcal disease). At minimum, those targeted diseases would have to be covered by our system.

Moreover, the advantage of the microsphere-based multiplex serological assays is the cost of the raw material expenses for production of microspheres coupled with antigens; in this study, the cost for 9 antigens per sample was about one US dollar including the cost for antigen preparations, purifications, coupling antigens with microspheres, and microsphere purchases. The low running cost for assays would be benefit to continue and geographically expand the surveillance activities, although we cannot conclude that the same cost can be applied if the manufacturing processes were transferred to a commercial basis. In contrast, the equipment (assay machine) for the microsphere-based multiplex serological assay is not inexpensive (about US$ 80,000). Because the manipulation of the device is simple and easy, we would encourage the establishment of a centralized system of the assays with a blood sampling framework from geographically wider areas; filter paper sampling or dried blood spot sampling would be a good option for such centralized surveillance system [44].

In summary, we report the development of a microsphere-based multiplex serological assay to simultaneously measure IgG against several antigens. This work is just the first step in the practical development and implementation of actual future programs. By adding pathogens and antigens of interest as well as geographically expanding the covered areas, we would be able to establish and optimize made-to-order high-quality programs with the ability to choose suitable pathogens and antigens according to the actual situations. This new assay system is expected to enable us to provide a monitoring framework that uses limited resources effectively to control NTDs and other infectious diseases in Africa.

## Supporting Information

Figure S1 **Sodium dodecyl sulfate polyacrylamide gel electrophoresis (SDS-PAGE) analysis of purified antigens.**
(PDF)

Figure S2 **Assay stability after coupling microspheres with antigens.**
(PDF)

Table S1 **BIC results of mixture models.**
(PDF)

Table S2 **Sensitivities and specificities in positive and negative controls using cut-off values calculated by the finite mixture model-based MFI value distributions in population-based serological survey.**
(PDF)

Table S3 **Seroprevalence by site, sex, and age group for pathogens measured simultaneously using a microsphere-based multi-serological assay on samples from the Kwale site.**
(PDF)

Table S4 **Seroprevalence by site, sex, and age group for pathogens measured simultaneously using a microsphere-based multi-serological assay on samples from the Mbita site.**
(PDF)

## References

[1] Hotez PJ, Kamath A (2009) Neglected tropical diseases in sub-saharan Africa: review of their prevalence, distribution, and disease burden. PLoS Negl Trop Dis 3: e412. 19707588 10.1371/journal.pntd.0000412 PMC2727001

[2] Department of Control of Neglected Tropical Diseases (2006) Neglected Tropical Diseases. Geneva: World Health Organization.

[3] Hotez PJ, Mistry N, Rubinstein J, Sachs JD (2011) Integrating neglected tropical diseases into AIDS, tuberculosis, and malaria control. N Engl J Med 364: 2086–2089. 21631320 10.1056/NEJMp1014637

[4] Solomon AW, Engels D, Bailey RL, Blake IM, Brooker S, et al (2012) A diagnostics platform for the integrated mapping, monitoring, and surveillance of neglected tropical diseases: rationale and target product profiles. PLoS Negl Trop Dis 6: e1746. 22860146 10.1371/journal.pntd.0001746 PMC3409112

[5] Vignali DA (2000) Multiplexed particle-based flow cytometric assays. J Immunol Methods 243: 243–255. 10986418 10.1016/s0022-1759(00)00238-6

[6] Khan IH, Ravindran R, Yee J, Ziman M, Lewinsohn DM, et al (2008) Profiling antibodies to Mycobacterium tuberculosis by multiplex microbead suspension arrays for serodiagnosis of tuberculosis. Clin Vaccine Immunol 15: 433–438. 18077619 10.1128/CVI.00354-07 PMC2268277

[7] Go YY, Wong SJ, Branscum AJ, Demarest VL, Shuck KM, et al (2008) Development of a fluorescent-microsphere immunoassay for detection of antibodies specific to equine arteritis virus and comparison with the virus neutralization test. Clin Vaccine Immunol 15: 76–87. 18032597 10.1128/CVI.00388-07 PMC2223870

[8] Clavijo A, Hole K, Li M, Collignon B (2006) Simultaneous detection of antibodies to foot-and-mouth disease non-structural proteins 3ABC, 3D, 3A and 3B by a multiplexed Luminex assay to differentiate infected from vaccinated cattle. Vaccine 24: 1693–1704. 16260073 10.1016/j.vaccine.2005.09.057

[9] Watson DS, Reddy SM, Brahmakshatriya V, Lupiani B (2009) A multiplexed immunoassay for detection of antibodies against avian influenza virus. J Immunol Methods 340: 123–131. 19000692 10.1016/j.jim.2008.10.007

[10] Moss DM, Priest JW, Boyd A, Weinkopff T, Kucerova Z, et al (2011) Multiplex bead assay for serum samples from children in Haiti enrolled in a drug study for the treatment of lymphatic filariasis. Am J Trop Med Hyg 85: 229–237. 21813840 10.4269/ajtmh.2011.11-0029 PMC3144818

[11] Griffin SM, Chen IM, Fout GS, Wade TJ, Egorov AI (2011) Development of a multiplex microsphere immunoassay for the quantitation of salivary antibody responses to selected waterborne pathogens. J Immunol Methods 364: 83–93. 21093445 10.1016/j.jim.2010.11.005

[12] Tachibana H, Cheng XJ, Masuda G, Horiki N, Takeuchi T (2004) Evaluation of recombinant fragments of Entamoeba histolytica Gal/GalNAc lectin intermediate subunit for serodiagnosis of amebiasis. J Clin Microbiol 42: 1069–1074. 15004055 10.1128/JCM.42.3.1069-1074.2004 PMC356887

[13] Takagi H, Islam MZ, Itoh M, Islam AU, Saifuddin Ekram AR, et al (2007) Short report: production of recombinant kinesin-related protein of Leishmania donovani and its application in the serodiagnosis of visceral leishmaniasis. Am J Trop Med Hyg 76: 902–905. 17488913

[14] Ishii K, Hisaeda H, Duan X, Imai T, Sakai T, et al (2006) The involvement of immunoproteasomes in induction of MHC class I-restricted immunity targeting Toxoplasma SAG1. Microbes and infection/Institut Pasteur 8: 1045–1053. 10.1016/j.micinf.2005.10.023 16515877

[15] Dissanayake S, Zheng H, Dreyer G, Xu M, Watawana L, et al (1994) Evaluation of a recombinant parasite antigen for the diagnosis of lymphatic filariasis. Am J Trop Med Hyg 50: 727–734. 8024066 10.4269/ajtmh.1994.50.727

[16] Gnann JW Jr, Schwimmbeck PL, Nelson JA, Truax AB, Oldstone MB (1987) Diagnosis of AIDS by using a 12-amino acid peptide representing an immunodominant epitope of the human immunodeficiency virus. J Infect Dis 156: 261–267. 2439614 10.1093/infdis/156.2.261

[17] Kulshrestha A, Gupta A, Verma N, Sharma SK, Tyagi AK, et al (2005) Expression and purification of recombinant antigens of Mycobacterium tuberculosis for application in serodiagnosis. Protein Expr Purif 44: 75–85. 15982900 10.1016/j.pep.2005.04.014

[18] Waterboer T, Sehr P, Pawlita M (2006) Suppression of non-specific binding in serological Luminex assays. J Immunol Methods 309: 200–204. 16406059 10.1016/j.jim.2005.11.008

[19] Pickering JW, Larson MT, Martins TB, Copple SS, Hill HR (2010) Elimination of false-positive results in a luminex assay for pneumococcal antibodies. Clin Vaccine Immunol 17: 185–189. 19923569 10.1128/CVI.00329-09 PMC2812081

[20] Infectious Disease Surveillance Center, National Institute of Infectious Diseases, Japan (2012) Annual Surveillance Data: Notifiable Diseases. Available: http://idsc.nih.go.jp/idwr/ydata/report-Ea.html. Accessed 17 April 2014.

[21] Kimura E, Itoh M (2011) Filariasis in Japan some 25 years after its eradication. Trop Med Health 39: 57–63. 22028603 10.2149/tmh.39-1-suppl_2-57 PMC3153153

[22] Nakamura-Uchiyama F, Hiromatsu K, Ishiwata K, Sakamoto Y, Nawa Y (2003) The current status of parasitic diseases in Japan. Intern Med 42: 222–236. 12705786 10.2169/internalmedicine.42.222

[23] Otsubo H, Yamaguchi K (2008) Current risks in blood transfusion in Japan. Jpn J Infect Dis 61: 427–433. 19050347

[24] Sakikawa M, Noda S, Hanaoka M, Nakayama H, Hojo S, et al (2012) Anti-Toxoplasma antibody prevalence, primary infection rate, and risk factors in a study of toxoplasmosis in 4,466 pregnant women in Japan. Clin Vaccine Immunol 19: 365–367. 22205659 10.1128/CVI.05486-11 PMC3294603

[25] Kaneko S, K'Opiyo J, Kiche I, Wanyua S, Goto K, et al (2012) Health and Demographic Surveillance System in the Western and Coastal Areas of Kenya: An Infrastructure for Epidemiologic Studies in Africa. J Epidemiol 22: 276–285. 22374366 10.2188/jea.JE20110078 PMC3798630

[26] Bentwich Z, Kalinkovich A, Weisman Z (1995) Immune activation is a dominant factor in the pathogenesis of African AIDS. Immunol Today 16: 187–191. 7734046 10.1016/0167-5699(95)80119-7

[27] Yazdanbakhsh M, Kremsner PG, van Ree R (2002) Allergy, parasites, and the hygiene hypothesis. Science 296: 490–494. 11964470 10.1126/science.296.5567.490

[28] Everitt BS (1996) An introduction to finite mixture distributions. Stat Methods Med Res 5: 107–127. 8817794 10.1177/096228029600500202

[29] Rota MC, Massari M, Gabutti G, Guido M, De Donno A, et al (2008) Measles serological survey in the Italian population: interpretation of results using mixture model. Vaccine 26: 4403–4409. 18585420 10.1016/j.vaccine.2008.05.094

[30] Baughman AL, Bisgard KM, Edwards KM, Guris D, Decker MD, et al (2004) Establishment of diagnostic cutoff points for levels of serum antibodies to pertussis toxin, filamentous hemagglutinin, and fimbriae in adolescents and adults in the United States. Clin Diagn Lab Immunol 11: 1045–1053. 15539504 10.1128/CDLI.11.6.1045-1053.2004 PMC524757

[31] Deb P (2007) FMM: Stata module to estimate finite mixture models. Available: http://ideas.repec.org/c/boc/bocode/s456895.html. Accessed 2 January 2014.

[32] Youden WJ (1950) Index for rating diagnostic tests. Cancer 3: 32–35. 15405679 10.1002/1097-0142(1950)3:1<32::aid-cncr2820030106>3.0.co;2-3

[33] Ngugi AK, Bottomley C, Kleinschmidt I, Wagner RG, Kakooza-Mwesige A, et al (2013) Prevalence of active convulsive epilepsy in sub-Saharan Africa and associated risk factors: cross-sectional and case-control studies. Lancet Neurol 12: 253–263. 23375964 10.1016/S1474-4422(13)70003-6 PMC3581814

[34] Marlet MV, Sang DK, Ritmeijer K, Muga RO, Onsongo J, et al (2003) Emergence or re-emergence of visceral leishmaniasis in areas of Somalia, north-eastern Kenya, and south-eastern Ethiopia in 2000-01. Trans R Soc Trop Med Hyg 97: 515–518. 15307414 10.1016/s0035-9203(03)80012-3

[35] Romero HD, Silva Lde A, Silva-Vergara ML, Rodrigues V, Costa RT, et al (2009) Comparative study of serologic tests for the diagnosis of asymptomatic visceral leishmaniasis in an endemic area. Am J Trop Med Hyg 81: 27–33. 19556562

[36] Yang B, Chen Y, Wu L, Xu L, Tachibana H, et al (2012) Seroprevalence of Entamoeba histolytica infection in China. Am J Trop Med Hyg 87: 97–103. 10.4269/ajtmh.2012.11-0626 PMC3391064 22764298

[37] Njenga SM, Mwandawiro CS, Wamae CN, Mukoko DA, Omar AA, et al (2011) Sustained reduction in prevalence of lymphatic filariasis infection in spite of missed rounds of mass drug administration in an area under mosquito nets for malaria control. Parasites & vectors 4: 90. 21612649 10.1186/1756-3305-4-90 PMC3125382

[38] Njomo DW, Amuyunzu-Nyamongo M, Magambo JK, Njenga SM (2012) The role of personal opinions and experiences in compliance with mass drug administration for lymphatic filariasis elimination in Kenya. PLoS One 7: e48395. 23185256 10.1371/journal.pone.0048395 PMC3501492

[39] Rao KV, Eswaran M, Ravi V, Gnanasekhar B, Narayanan RB, et al (2000) The Wuchereria bancrofti orthologue of Brugia malayi SXP1 and the diagnosis of bancroftian filariasis. Mol Biochem Parasitol 107: 71–80. 10717303 10.1016/s0166-6851(99)00231-5

[40] Levine MM, Young CR, Black RE, Takeda Y, Finkelstein RA (1985) Enzyme-linked immunosorbent assay to measure antibodies to purified heat-labile enterotoxins from human and porcine strains of Escherichia coli and to cholera toxin: application in serodiagnosis and seroepidemiology. J Clin Microbiol 21: 174–179. 3882744 10.1128/jcm.21.2.174-179.1985 PMC271608

[41] Kotloff KL, Nataro JP, Blackwelder WC, Nasrin D, Farag TH, et al (2013) Burden and aetiology of diarrhoeal disease in infants and young children in developing countries (the Global Enteric Multicenter Study, GEMS): a prospective, case-control study. Lancet 382: 209–222. 23680352 10.1016/S0140-6736(13)60844-2

[42] Qadri F, Svennerholm AM, Faruque AS, Sack RB (2005) Enterotoxigenic Escherichia coli in developing countries: epidemiology, microbiology, clinical features, treatment, and prevention. Clin Microbiol Rev 18: 465–483. 16020685 10.1128/CMR.18.3.465-483.2005 PMC1195967

[43] Ministry of Public Health and Sanitation; Republic of Kenya (2011) National Multi-year Strategic Plan for control of Neglected Tropical diseases 2011–1015.

[44] Lammie PJ, Moss DM, Brook Goodhew E, Hamlin K, Krolewiecki A, et al (2012) Development of a new platform for neglected tropical disease surveillance. Int J Parasitol 42: 797–800. 22846784 10.1016/j.ijpara.2012.07.002

