# Supplementary material for: Serological Surveillance Development for Tropical Infectious Diseases Using Simultaneous Microsphere-Based Multiplex Assays and Finite Mixture Models
Source: PLoS Negl Trop Dis. 2014 Jul 31;8(7):e3040. doi: 10.1371/journal.pntd.0003040 (PMC4117437; doi:10.1371/journal.pntd.0003040)
Supplement: Figure S1 — Sodium dodecyl sulfate polyacrylamide gel electrophoresis (SDS-PAGE) analysis of purified antigens. (PDF) [file pntd.0003040.s001.pdf]

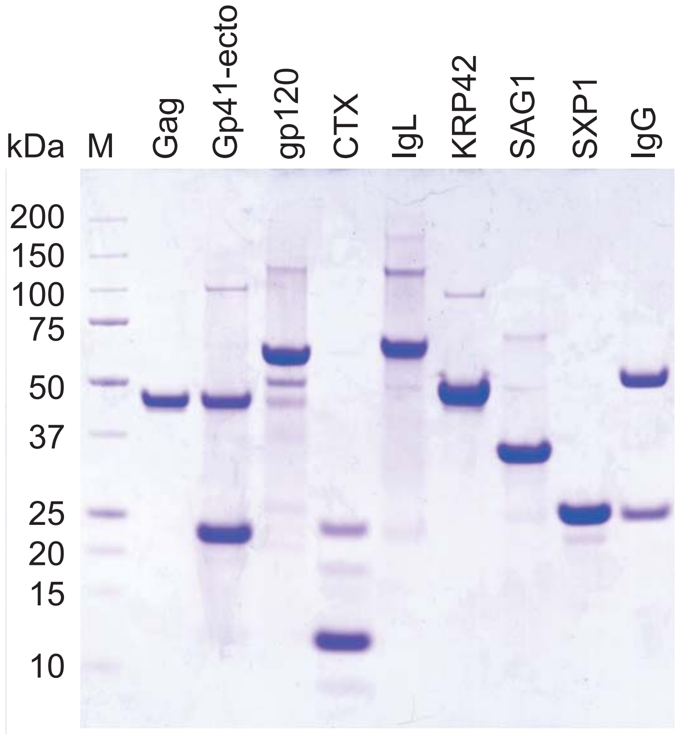

Figure S1. Sodium dodecyl sulfate polyacrylamide gel electrophoresis (SDS-PAGE) analysis of purified antigens
