# Supplementary material for: Serological Surveillance Development for Tropical Infectious Diseases Using Simultaneous Microsphere-Based Multiplex Assays and Finite Mixture Models
Source: PLoS Negl Trop Dis. 2014 Jul 31;8(7):e3040. doi: 10.1371/journal.pntd.0003040 (PMC4117437; doi:10.1371/journal.pntd.0003040)
Supplement: Figure S2 — Assay stability after coupling microspheres with antigens. (PDF) [file pntd.0003040.s002.pdf]

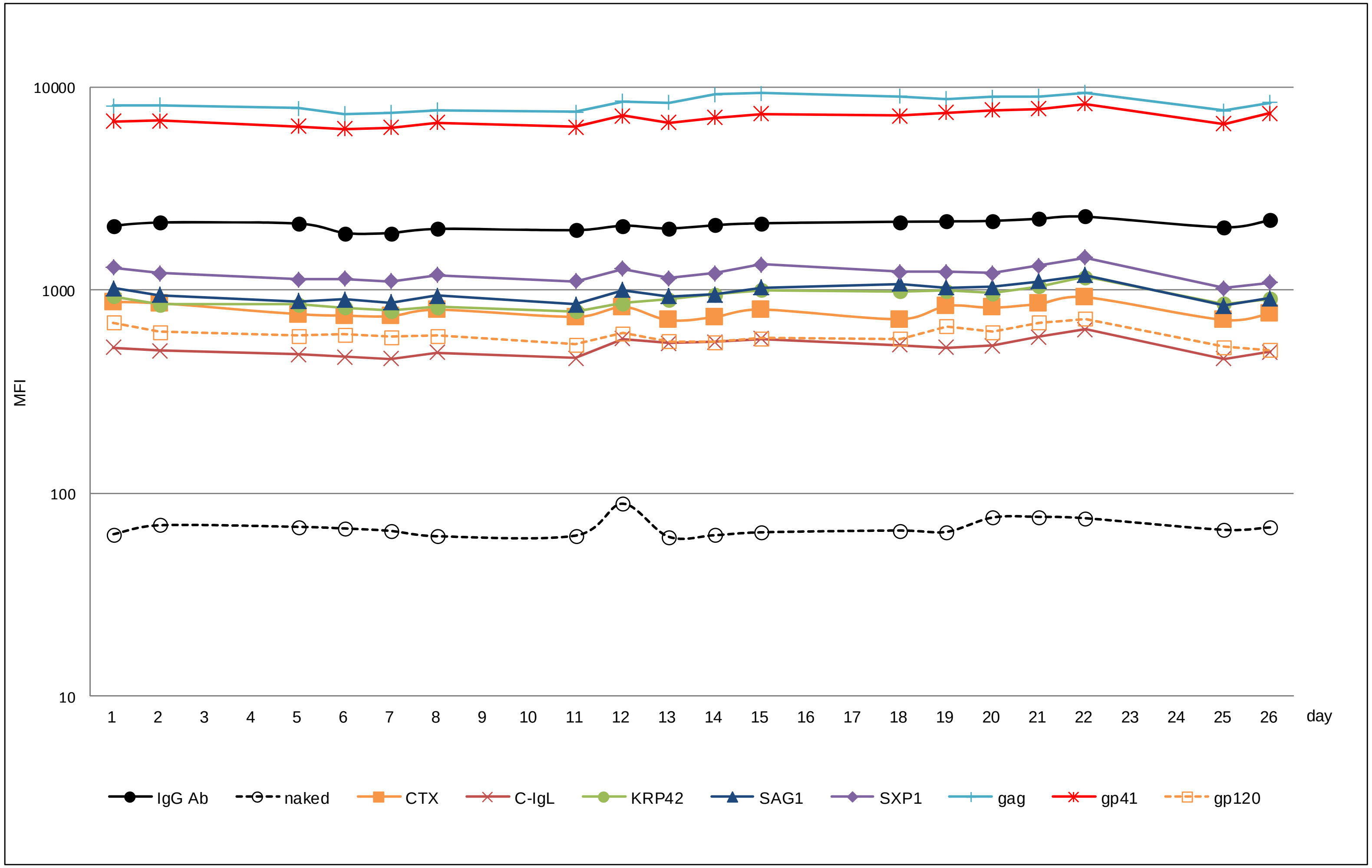

Figure S2. Assay stability after coupling microspheres with antigens

Plotted average median fluorescence intensity (MFI) of mixed positive serums; one to four plates per day assayed during 26 days, but not daily; coupled microspheres with antigens stored at 4°C
