# Supplementary material for: Serological Surveillance Development for Tropical Infectious Diseases Using Simultaneous Microsphere-Based Multiplex Assays and Finite Mixture Models
Source: PLoS Negl Trop Dis. 2014 Jul 31;8(7):e3040. doi: 10.1371/journal.pntd.0003040 (PMC4117437; doi:10.1371/journal.pntd.0003040)
Supplement: Table S1 — BIC results of mixture models. (PDF) [file pntd.0003040.s003.pdf]

Table S1. BIC and number of components obtained by finite mixture model

Pathogen	Antigen	number of components	AIC (Akaike information criterion)	BIC (Bayesian information criterion)
HIV	gp41	2	-2204.037	-2173.363
		3	-2939.859	-2890.781
		**4**	**-3089.633**	**-3022.151**
		5	-3104.587	-3018.701
	gp120	2	-133.1108	-102.437
		3	-391.6685	-342.5904
		**4**	**-456.9757**	**-389.4933**
		5	-454.4575	-368.5708
	gag	2	1585.118	1615.792
		3	954.8943	1003.972
		4	776.1338	843.6162
		**5**	**712.6383**	**798.5249**
		6	700.6908	804.9817
W. bancrofti	SXP1	2	1599.353	1630.027
		**3**	**1552.889**	**1601.967**
		4	1537.351	1604.833
L. donovani	KRP42	2	3353.388	3384.062
		**3**	**3313.9**	**3362.978**
		4	3299.853	3367.335
E. histolytica	C-IgL	2	297.1231	327.797
		**3**	**183.576**	**232.6541**
		4	165.3139	232.7962
Cholera Toxin	CTX	**2**	**-690.5053**	**-659.8315**
		3	-695.2258	-646.1477
T. gondii	SAG1	2	5242.179	5272.853
		3	4657.758	4706.836
		4	4520.948	4588.43
		**5**	**4482.768**	**4568.654**
		6	4477.132	4581.423
