# Supplementary material for: Serological Surveillance Development for Tropical Infectious Diseases Using Simultaneous Microsphere-Based Multiplex Assays and Finite Mixture Models
Source: PLoS Negl Trop Dis. 2014 Jul 31;8(7):e3040. doi: 10.1371/journal.pntd.0003040 (PMC4117437; doi:10.1371/journal.pntd.0003040)
Supplement: Table S2 — Sensitivities and specificities in positive and negative controls using cut-off values calculated by the finite mixture model-based MFI value distributions in population-based serological survey. (PDF) [file pntd.0003040.s004.pdf]

Table S2. Sensitivities and specificities in positive and negative controls using cutoff values calculated by the finite mixture model based MFI value distributions in population-based serological survey

Pathogen	Antigen	Cutoff value	Positive control			Negative control			Sensitivity		Specificity	
			Total	Positives	False negatives	Total	Positives	False positives	%	95%CI	%	95%CI
HIV1	gp41	1552.4	50	50	0	40	0	0	100.0%	(92.9 - 100)	100.0%	(91.2 - 100)
	gag	257.0	50	40	10	40	0	0	80.0%	(66.3 - 90)	100.0%	(91.2 - 100)
	gp120	420.7	50	36	14	40	0	0	72.0%	(57.5 - 83.8)	100.0%	(91.2 - 100)
V. cholerae	CTX	106.9	7	7	0	40	11	11	100.0%	(59 - 100)	72.5%	(56.1 - 85.4)
E. histolytica	C-IgL	217.3	20	20	0	40	0	0	100.0%	(83.2 - 100)	100.0%	(91.2 - 100)
L. donovani	KRP42	405.5	16	15	1	40	0	0	93.8%	(69.8 - 99.8)	100.0%	(91,2 - 100)
W. bancrofti	SXP1*	358.9	20	20	0	40	3	3	100.0%	(83.2 - 100)	92.5%	(79.6 - 98.4)
T. gondii	SAG1	608.1	19	18	1	40	0	0	94.7%	(74 - 99.9)	100.0%	(91.2 - 100)

* The cutoff value between the second highest and third highest normal distributions divided by finite mixture mode was used. For others the highest and second highest normal distributions were used for calculating cutoff values.
