# Supplementary material for: Serological Surveillance Development for Tropical Infectious Diseases Using Simultaneous Microsphere-Based Multiplex Assays and Finite Mixture Models
Source: PLoS Negl Trop Dis. 2014 Jul 31;8(7):e3040. doi: 10.1371/journal.pntd.0003040 (PMC4117437; doi:10.1371/journal.pntd.0003040)
Supplement: Table S3 — Seroprevalence by site, sex, and age group for pathogens measured simultaneously using a microsphere-based multi-serological assay on samples from the Kwale site. (PDF) [file pntd.0003040.s005.pdf]

Table S3. Age and sex specific seropositivities for each pathogen in Kwale site

pathogen	sex	age 0-4 y/o	age 5-9 y/o	age 10-14 y/o	age 15-19 y/o	age 20-24 y/o	age 25-29 y/o	age 30-34 y/o	age 35-39 y/o	age 40-44 y/o	over 45 y/o	Total
HIV	female	0.0%	1.2%	0.0%	1.5%	1.7%	5.1%	6.5%	5.1%	12.7%	3.7%	3.7%
	male	1.1%	0.0%	1.2%	3.9%	1.8%	0.0%	1.9%	3.6%	6.0%	4.2%	2.3%
W. bancrofti	female	14.3%	17.1%	4.5%	16.7%	15.5%	22.0%	15.6%	25.3%	27.8%	34.6%	19.2%
	male	15.7%	17.0%	14.3%	10.5%	28.6%	18.2%	28.8%	30.9%	43.3%	49.3%	24.7%
L. donovani	female	11.0%	13.4%	9.1%	18.2%	12.1%	5.1%	7.8%	6.3%	15.2%	12.3%	11.1%
	male	10.1%	17.0%	11.9%	13.2%	16.1%	18.2%	13.5%	9.1%	14.9%	19.7%	14.3%
E. histolytica	female	7.7%	15.9%	14.8%	16.7%	10.3%	10.2%	18.2%	12.7%	8.9%	9.9%	12.5%
	male	6.7%	15.9%	14.3%	9.2%	7.1%	21.8%	11.5%	14.5%	11.9%	19.7%	13.1%
V. Cholerae	female	72.5%	45.1%	25.0%	18.2%	19.0%	25.4%	19.5%	17.7%	21.5%	19.8%	29.6%
	male	76.4%	46.6%	20.2%	23.7%	16.1%	27.3%	21.2%	25.5%	29.9%	31.0%	33.9%
T. gondii	female	3.3%	7.3%	17.0%	30.3%	25.9%	30.5%	41.6%	53.2%	54.4%	59.3%	31.8%
	male	2.2%	14.8%	21.4%	21.1%	30.4%	30.9%	28.8%	45.5%	53.7%	67.6%	29.9%
