# Supplementary material for: Serological Surveillance Development for Tropical Infectious Diseases Using Simultaneous Microsphere-Based Multiplex Assays and Finite Mixture Models
Source: PLoS Negl Trop Dis. 2014 Jul 31;8(7):e3040. doi: 10.1371/journal.pntd.0003040 (PMC4117437; doi:10.1371/journal.pntd.0003040)
Supplement: Table S4 — Seroprevalence by site, sex, and age group for pathogens measured simultaneously using a microsphere-based multi-serological assay on samples from the Mbita site. (PDF) [file pntd.0003040.s006.pdf]

Table S4. Age and sex specific seropositivities for each pathogen in Mbita site

pathogen	sex	age 0-4 y/o	age 5-9 y/o	age 10-14 y/o	age 15-19 y/o	age 20-24 y/o	age 25-29 y/o	age 30-34 y/o	age 35-39 y/o	age 40-44 y/o	over 45 y/o	Total
HIV	female	7.8%	6.6%	2.9%	8.1%	25.6%	34.0%	48.5%	45.1%	39.8%	25.0%	24.1%
	male	3.1%	4.7%	4.8%	3.0%	4.7%	17.2%	34.8%	35.6%	35.2%	20.7%	15.8%
W. bancrofti	female	16.7%	20.8%	16.2%	16.2%	10.5%	6.0%	11.3%	7.8%	6.8%	13.0%	12.6%
	male	17.5%	18.7%	19.0%	15.8%	11.8%	15.1%	13.0%	8.0%	14.3%	9.8%	14.5%
L. donovani	female	19.6%	31.1%	28.6%	21.2%	9.3%	14.0%	15.5%	12.7%	7.8%	11.1%	17.3%
	male	23.7%	22.4%	24.8%	11.9%	21.2%	17.2%	8.7%	10.3%	18.7%	13.0%	17.4%
E. histolytica	female	22.5%	22.6%	24.8%	18.2%	16.3%	13.0%	13.4%	9.8%	12.6%	18.5%	17.3%
	male	21.6%	20.6%	21.9%	18.8%	17.6%	12.9%	17.4%	10.3%	6.6%	8.7%	15.9%
V. Cholerae	female	56.9%	32.1%	22.9%	24.2%	19.8%	16.0%	12.4%	19.6%	15.5%	21.3%	24.2%
	male	55.7%	32.7%	29.5%	20.8%	27.1%	23.7%	17.4%	13.8%	11.0%	21.7%	25.7%
T. gondii	female	10.8%	14.2%	18.1%	16.2%	36.0%	35.0%	38.1%	36.3%	50.5%	58.3%	31.3%
	male	5.2%	15.0%	22.9%	20.8%	20.0%	19.4%	28.3%	32.2%	35.2%	54.3%	24.9%
